# Decomposing solar and geomagnetic activity and seasonal dependencies to examine the relationship between GPS loss of lock and ionospheric turbulence

**DOI:** 10.1038/s41598-023-34727-2

**Published:** 2023-06-07

**Authors:** Giulia Lovati, Paola De Michelis, Giuseppe Consolini, Michael Pezzopane, Alessio Pignalberi, Francesco Berrilli

**Affiliations:** 1 Dipartimento di Fisica, Universitá di Roma Sapienza, 00185 Rome, Italy; 2 Istituto Nazionale di Geofisica e Vulcanologia, 00143 Rome, Italy; 3 INAF-Istituto di Astrofisica e Planetologia Spaziali, 00133 Rome, Italy; 4 Dipartimento di Fisica, Universitá di Roma Tor Vergata, 00133 Rome, Italy

**Keywords:** Space physics, Physics

## Abstract

Ionospheric irregularities are plasma density variations that occur at various altitudes and latitudes and whose size ranges from a few meters to a few hundred kilometers. They can have a negative impact on the Global Navigation Satellite Systems (GNSS), on their positioning accuracy and even cause a signal loss of lock (LoL), a phenomenon for which GNSS receivers can no longer track the satellites’ signal. Nowadays, the study of plasma density irregularities is important because many of the crucial infrastructures of our society rely on the efficient operation of these positioning systems. It was recently discovered that, of all possible ionospheric plasma density fluctuations, those in a turbulent state and characterized by extremely high values of the Rate Of change of the electron Density Index appear to be associated with the occurrence of LoL events. The spatial distributions of this class of fluctuations at mid and high latitudes are reconstructed for the first time using data collected on Swarm satellites between July 15th, 2014 and December 31st, 2021, emphasizing their dependence on solar activity, geomagnetic conditions, and season. The results unequivocally show that the identified class of plasma fluctuations exhibits spatio-temporal behaviours similar to those of LoL events.

## Introduction

Global Navigation Satellite Systems (GNSS) are becoming increasingly important to our society. The efficient operation of GNSS is essential for many of our vital infrastructures, including for instance power systems, railway control, sea transport, civil and military aircraft, communication technologies, government services, banking and finance systems^1^. The critical importance of a well-performing GNSS is highlighted when the degree of complexity of the strategic infrastructure interdependence in our modern society is considered. One of the natural factors that degrades GNSS signals as they propagate through the ionosphere, lowering their accuracy and reliability, is the presence of plasma irregularities^2^. Indeed, these can affect the propagation of electromagnetic signals through the ionosphere, causing their random scattering and, in some cases, their total reflection. In our modern society, it is critical to understand the causes of ionospheric irregularities and monitor their dynamics, particularly during space weather events when the number of ionospheric irregularities tends to increase significantly.

What is currently known is that the ionospheric irregularities are caused by plasma density fluctuations ranging from a few meters to hundreds of kilometers, and that they are affected by both geophysical parameters such as spatial coordinates, local time, and season, as well as solar activity. Plasma density irregularities, in particular, increase during periods of high solar activity and have a greater impact especially at equatorial and high latitudes, while their number and influence is limited in the mid-latitude region^3^. Also geomagnetic activity has a large influence on irregularities, especially in high-latitude areas where substorm conditions favor them^4^. Furthermore, they appear to be connected to large-scale plasma structures like polar patches, cusp aurora, and auroral blobs, whose evolution and position depend on both the solar wind and the polarity and strength of the interplanetary magnetic field^5,6^.

With the launch of the Swarm mission, there was a new opportunity to track the amplitudes, variability, and scaling invariance properties of the plasma irregularities in the ionospheric F-region along the satellite tracks for more than half of the 24th solar cycle and, at this time, for the first months of the 25th. This allows investigating plasma density irregularities and their effects on Swarm Global Positioning System (GPS) receivers, which may result in a signal degradation or, in the worst-case scenario, a signal interruption when a loss of lock (LoL) event is occurring. In detail, Swarm LoL events have been thoroughly investigated in order to determine the types of irregularities and background conditions that cause them^7^, as well as their dependencies on geographic and magnetic coordinates, geophysical conditions, and solar activity^8–11^.

It is known that some ionospheric irregularities can be characterized by turbulent processes^3,12,13^ triggered by various plasma instabilities that affect both their formation and evolution. Indeed, some plasma density irregularities at high latitudes, particularly in the polar regions, are produced by the gradient-drift instability^14,15^, which is brought on the differential drifts of ions and electrons on a plasma density gradient, by the Kelvin-Helmholtz instability, which occurs in the presence of strong flow shears^16,17^, and by the $\vec {E}\times \vec {B}$ instability^18^; instabilities that can evolve toward a nonlinear regime. The nonlinear development of plasma irregularities and their characteristic density power spectrum shape, which indicates scale invariance, recall what was developed in the context of fluid turbulence theory. It is imaginable that, under certain circumstances, the flow of ionospheric plasma can be characterized by the development of vortexes of various sizes and lifetimes, leading to chaotic and unpredictable motions present across a wide range of spatial scales, analogously to a turbulent fluid where the energy cascade process from large to small scales is described by the power law for the velocity field^19^.

Ionospheric electron density power spectra have been the subject of numerous studies, with spectral index values ranging from 1.5 to 2.5, depending on the instrumentation used, the particular ionospheric region being studied, and the range of scale lengths being analyzed^20–24^. Recently, by analyzing in situ 1 Hz rate electron density measurements from the European Space Agency (ESA) Swarm A satellite, interesting results have been attained within this framework. These studies have shown that there is a specific class of plasma density irregularities in the topside ionosphere, the origin of which appears to be related to the occurrence of turbulent processes^13,25,26^. This class is also consistently connected to extremely high values of the Rate Of change of electron Density Index (RODI), a proxy for the intensity of the fluctuations that characterize the ionospheric medium. Subsequent findings indicate that this particular class of $N_\mathrm{e}$ fluctuations could be related to GPS LoL events identified onboard Swarm A and Swarm B satellites^7^. These findings have highlighted the statistical relationship between LoL events and specific ionospheric plasma conditions, but without establishing whether these specific plasma density fluctuations actually exhibit spatio-temporal behaviors similar to LoL events. This type of testing is necessary to determine whether the detection of an electron density fluctuation with specific properties can be used as a proxy for LoL events. As a result, a comparison of the properties of LoL events and those of the identified plasma density fluctuations as a function of magnetic latitude, magnetic local time (MLT), geomagnetic activity, local season, and solar activity is required. Understanding whether this class of plasma density fluctuations can actually be used as a proxy for LoL events might pave the way for a future development of empirical models that can pinpoint the areas where specific physical conditions could trigger disturbances potentially harmful to radio signal propagation.

## Data

Data used in this work were collected by the Langmuir probes^27^ and the precise orbit determination (POD) antennas^28^ on board two of the three satellites of Swarm constellation, Swarm A and Swarm B^29^. Both satellites have a near-polar orbit, but while for Swarm A the initial altitude was at about 460 km, for Swarm B was approximately 510 km. Each of the Swarm satellites is outfitted with the same set of instruments which measure the electric and magnetic fields, as well as plasma density and temperature. In this work we analysed the electron density ($N_\mathrm{e}$) and the Total Electron Content (TEC) time-series, measured by Langmuir probes and POD antennas, respectively. Both time-series have a resolution of 1 Hz and are publicly distributed. They have been downloaded from the ESA dissemination server (ftp://swarm-diss.eo.esa.int).

From $N_\mathrm{e}$ time-series, RODI was calculated. This index is defined as the standard deviation of $N_\mathrm{e}$ time derivative, calculated on a sliding window of fixed width ($\Delta t=10$ s; for more information, see Pignalberi, 2021^30^). This time-series has a resolution of 1 Hz as well.

From the slant TEC (sTEC) data, the GPS LoL events were identified just by looking for interruptions in the sTEC time series. We only considered interruptions with a maximum length of 1200 s, in order to select interruptions that could really identify LoL events. By making this decision, it was ensured that the identified interruptions were not caused by the satellite leaving the field of view^11^. We only took into consideration events lasting longer than 1 s when determining the minimum length of interruptions in the sTEC time series. The latter decision was made since it appeared that the majority of LoL occurrences with a length of 1 s were not actually real^11^. It is worth highlighting that, using Level-2 Swarm TEC data^31^, the number of recorded LoL events is lower than what could have been obtained using Level-1B Swarm RINEX GPS data. This is because sTEC data are provided only for elevation angles greater than $20^{\circ }$.

Lastly, we used the scaling exponent values of the second-order structure function as a metric capable of identifying potential turbulent processes in the plasma density at the base of GPS LoL events. This quantity, as described in the “Methods” section, can be used to infer the spectral features of the analyzed time series, allowing a differentiation of the various types of instabilities and turbulent processes that caused the observed fluctuations.

**Figure 1:**
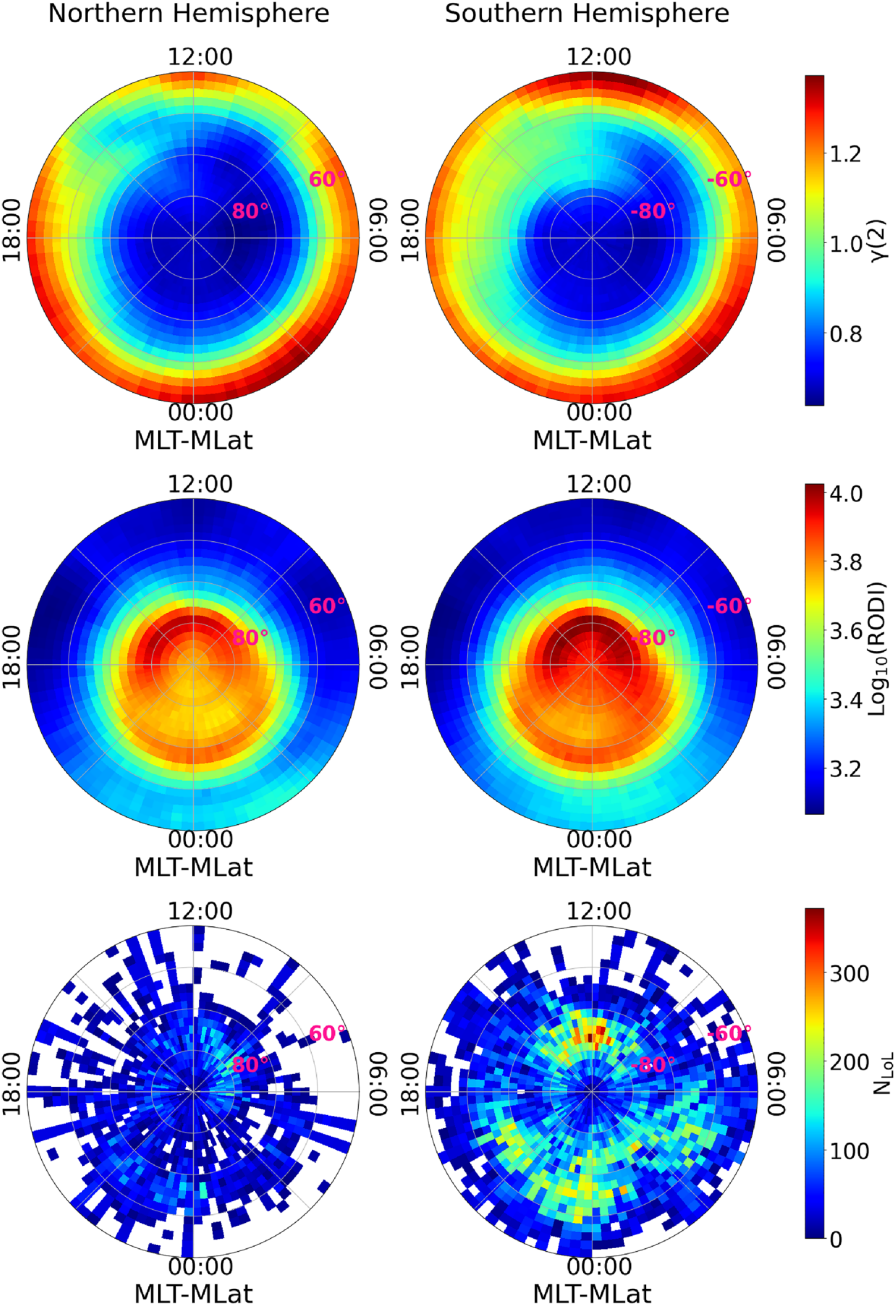
From top to bottom: polar view maps of the mean second-order scaling exponent ($\gamma (2$)) values, the mean RODI and the number of GPS LoL events, as identified using data recorded from July 15th, 2014 to December 31st, 2021 onboard Swarm A and Swarm B, in the mid- and high-latitude region ($|\textrm{MLat}| > 50^\circ$) of the Northern (left column) and Southern hemisphere (right column), respectively. Maps are in MLat and MLT reference frame. The data are binned in cells of $2^\circ$ in latitude and $4^\circ$ in MLT.

Figure 1 depicts all of the physical quantities that support the analysis presented in this study. In detail, it reports polar view maps of the mean second-order scaling exponent ($\gamma (2$)) values, the mean RODI and the number of GPS LoL events, as identified using data recorded from July 15th, 2014 to December 31st, 2021 on board Swarm A and Swarm B, for the Northern and Southern hemisphere, respectively. The coordinate system for all maps is Quasi-Dipole (QD) magnetic latitude (hereafter referred as MLat for brevity) and MLT^32–34^, with noon at the top and midnight at the bottom, and a binning grid of $2^{\circ } \times 4^{\circ }$ respectively, where $4^{\circ }$ corresponds to 16 min in MLT. These maps demonstrate that, depending on MLat, MLT, and hemisphere, $N_\mathrm{e}$ fluctuations are characterized by different values of the second-order scaling exponent and RODI. It is important to note that none of these quantities, taken individually, exhibit characteristics similar to the distribution of LoL events.

## Results

**Figure 2:**
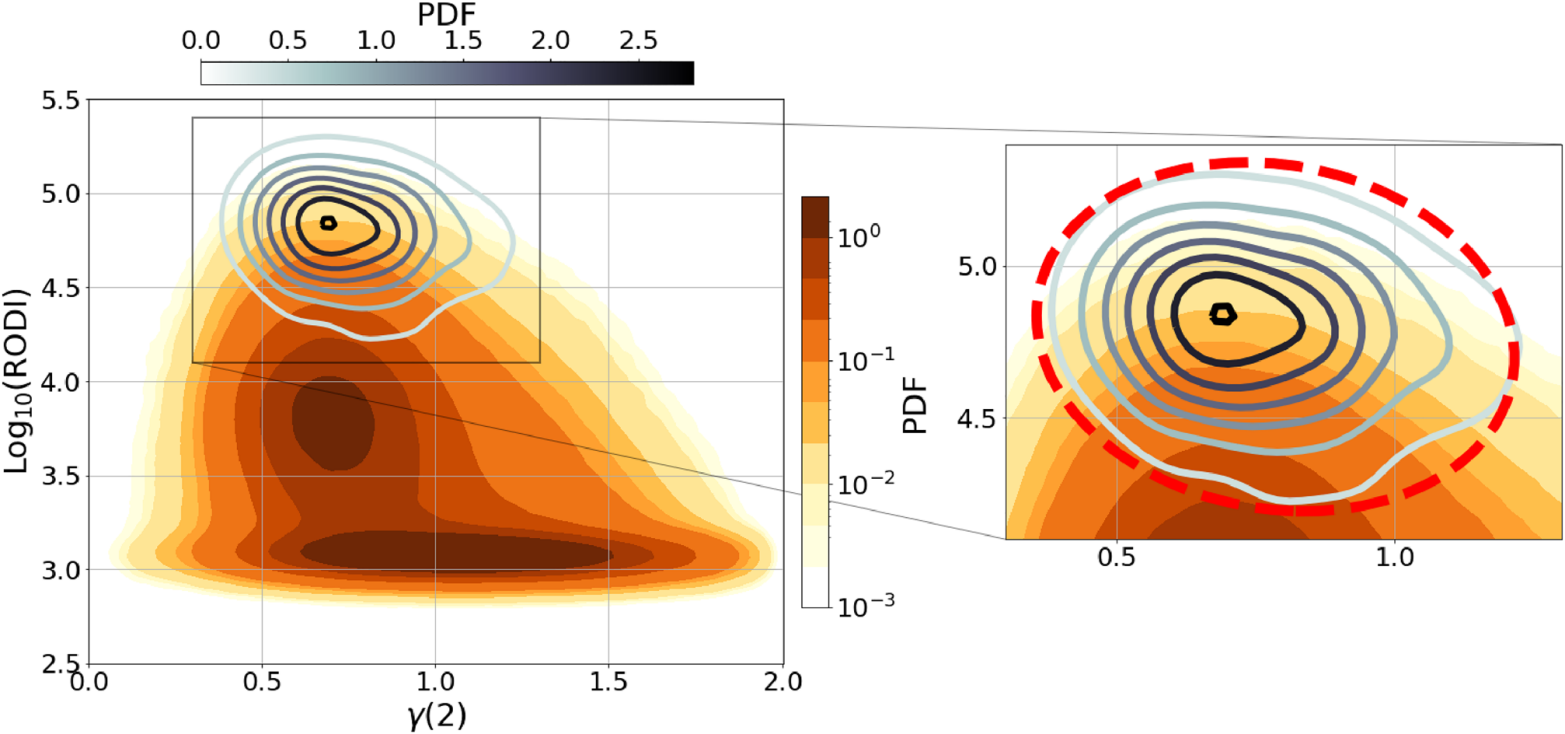
Conditioned joint probability density function (PDF) of RODI and $\gamma (2)$ in the mid- and high-latitude regions ($|\textrm{MLat}|>50^{\circ }$), considering all data recorded from July 15th, 2014 to December 31st, 2021 onboard Swarm A and Swarm B (yellow scale). Superposed the same quantity obtained considering data corresponding to the loss of lock events (grey-scale line contours). On the right a magnification of the figure on the left, where the red dashed curve identifies the ellipse obtained minimizing the sum of the squares of the residuals from the points of probability density equal to a tenth of the maximum.

Recently, it has been shown^7^ that the occurrence of GPS LoL events is linked to $N_\mathrm{e}$ fluctuations with extraordinarily high RODI values ($>10^4$ cm$^{-3}$ s$^{-1}$) and a second-order scaling exponent $\gamma (2)\le 1$. Figure 2 shows these findings. It displays the joint probability density function of RODI and $\gamma (2)$ during the selected period (from July 15th, 2014 to December 31st, 2021) for the complete dataset at mid and high latitudes, as yellow-scale filled contours, and the one conditioned to the occurrence of GPS LoL events, represented by grey-scale line contours. In this case, neither the two hemispheres nor the two chosen satellites are distinguished from one another.

According to previous analyses^7,25,35^, this result suggests the existence of two classes of $N_\mathrm{e}$ fluctuations, each with a different mean value for the second-order scaling exponents as well as the ionospheric index under consideration, implying that various instabilities and turbulent processes may be at work in causing the observed scaling features. The energy spectrum of only one of these classes of $N_\mathrm{e}$ fluctuations provides evidence that turbulent events contributed to their creation. Furthermore, this class is always associated with extremely high RODI values. This implies that, of all possible ionospheric irregularities, those caused by turbulent processes appear to be accompanied by $N_\mathrm{e}$ fluctuations that are stronger than those caused by other sources. This particular kind of $N_\mathrm{e}$ fluctuations is linked to the occurrence of GPS LoL events.

Here, we investigate the possibility of using $N_\mathrm{e}$ fluctuations that seem to be at the origin of GPS LoL events as a proxy for their occurrence. To achieve this, we select the values of the $\gamma (2)$-RODI associated with the GPS LoL occurrence. Given the shape of line contours of the PDF of $\gamma (2)$-RODI associated with the GPS LoL occurrence (grey-scale line contours), we use the least squares method to fit an ellipse to the curve corresponding to $\frac{1}{10}$ of the probability density maximum. The resulting ellipse is represented by the red dashed curve in the right panel of Fig. 2. In this manner, we obtained a new subset of Swarm data, corresponding to all those instants of time during which $\gamma (2)$ and RODI together were inside this curve, satisfying what we can call the ellipse’s criterion for brevity. This lists all the events that, regardless of whether a LoL event actually occurred, have values for $\gamma (2)$ and RODI that are potentially capable of triggering one. The GPS LoL event distribution is also conditioned in the following analysis to meet the ellipse’s criterion. This primarily removes from the distribution some of the already few events occurring in years of low solar activity, as well as the final portion of some longer-duration events.

**Figure 3:**
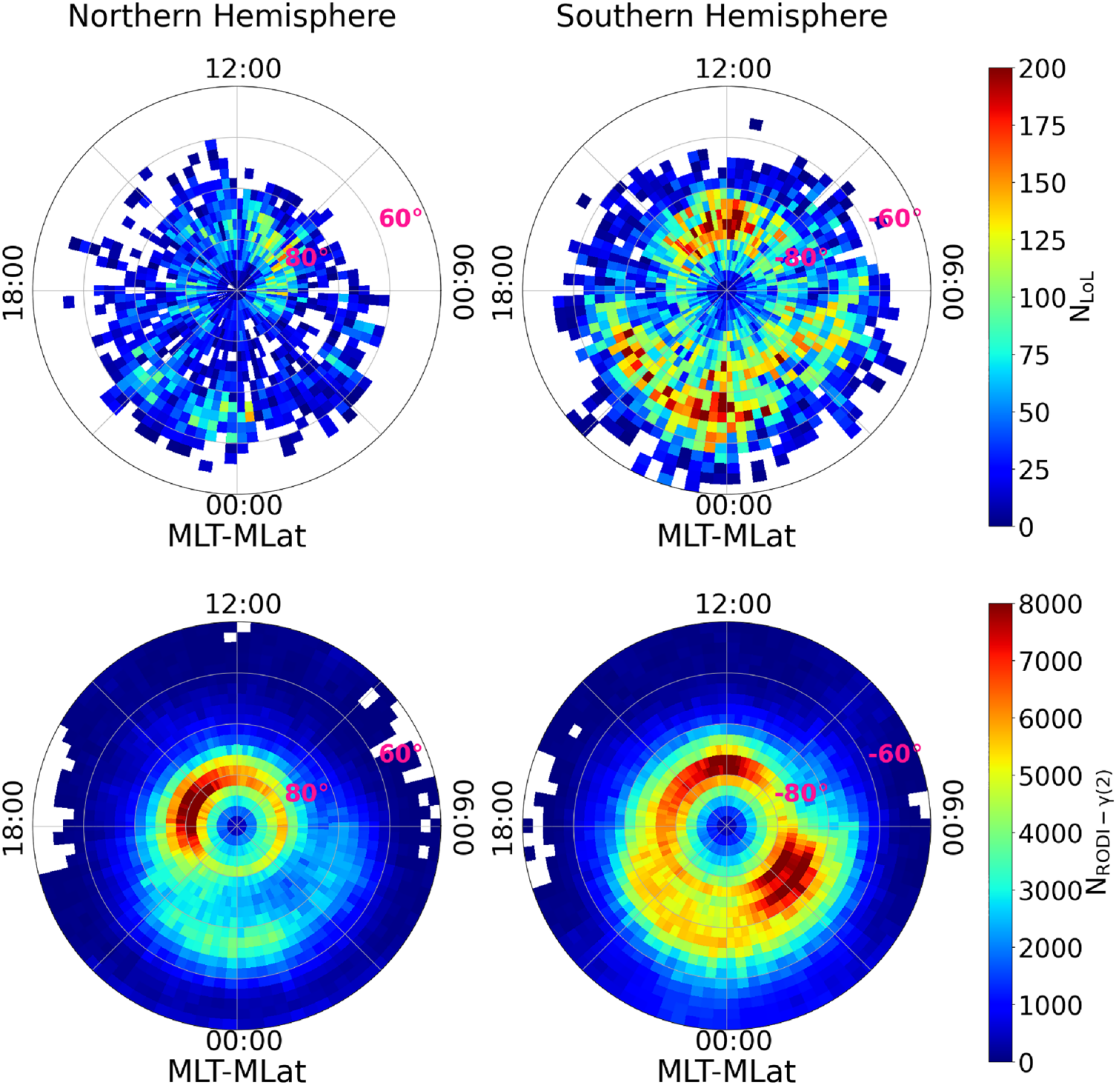
Comparison of the spatial distribution of GPS LoL events at mid and high latitudes ($|\textrm{MLat}|>50^{\circ }$) in the Northern and Southern hemispheres and the spatial distribution of $N_\mathrm{e}$ fluctuations as defined by $\gamma (2)$ and RODI values within the ellipse identified in Fig. 2.

Figure 3 compares the spatial distribution of GPS LoL events at mid and high latitudes ($|\textrm{MLat}|>50^{\circ }$) in the Northern and Southern hemispheres, as well as the spatial distribution of $N_\mathrm{e}$ fluctuations as defined by $\gamma (2)$ and RODI values within the ellipse. There is a high degree of agreement between the two distributions in both hemispheres. The occurrence of GPS LoL events is generally low below $|60^{\circ }|$ of MLat. It increases in the dayside range $|70^{\circ }-80^{\circ }|$ of MLat, which corresponds to the position of the cusp, and in the nigthside auroral oval between 18:00 and 06:00 MLT. Lastly, there are many more GPS LoL events in the Southern hemisphere than in the Northern one. The spatial distribution of $N_\mathrm{e}$ fluctuations with $\gamma (2)$ and RODI values within the ellipse exhibits similar patterns.

**Figure 4:**
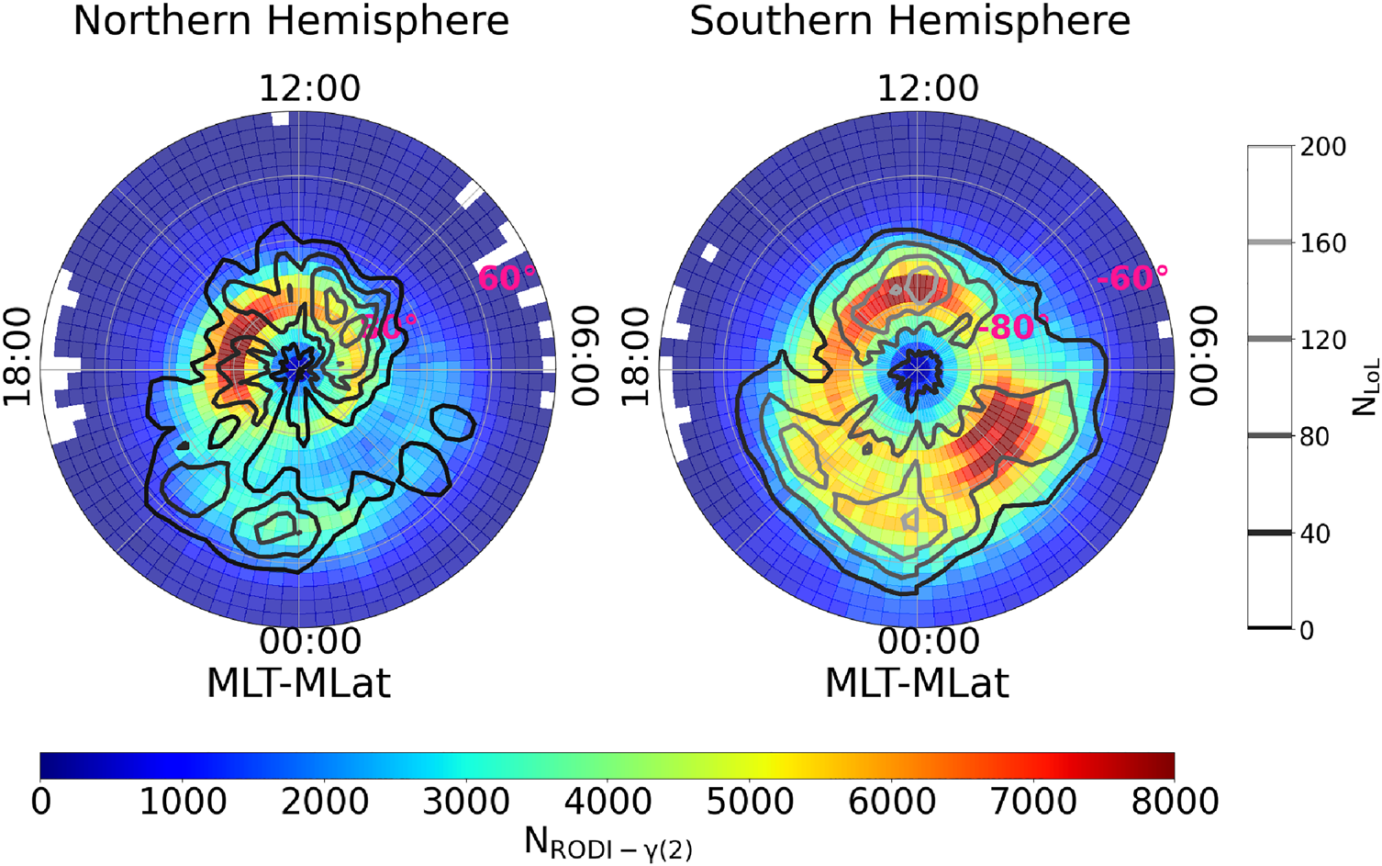
Superposition of the spatial distribution of the locations where $N_\mathrm{e}$ fluctuations exhibit $\gamma (2)$ and RODI values consistent with the occurrence of GPS LoL events (colored scale) and the spatial distribution of GPS LoL events (grey-scale line contours) at mid and high latitudes ($|\textrm{MLat}|>50^{\circ }$) in the Northern and Southern hemispheres.

Figure 4 shows the superposition of the two spatial distributions shown in Fig. 3. In this case the spatial distribution of GPS LoL events is reported using grey-scale contour lines. The good agreement between the two spatial distributions demonstrates that ionospheric $N_\mathrm{e}$ fluctuations with particular characteristics are primarily responsible for the development of GPS LoL events. The only significant difference is the number of GPS LoL events which is by far lower than the number of $N_\mathrm{e}$ fluctuations with values for $\gamma (2)$ and RODI potentially capable of producing GPS LoL events.

**Figure 5:**
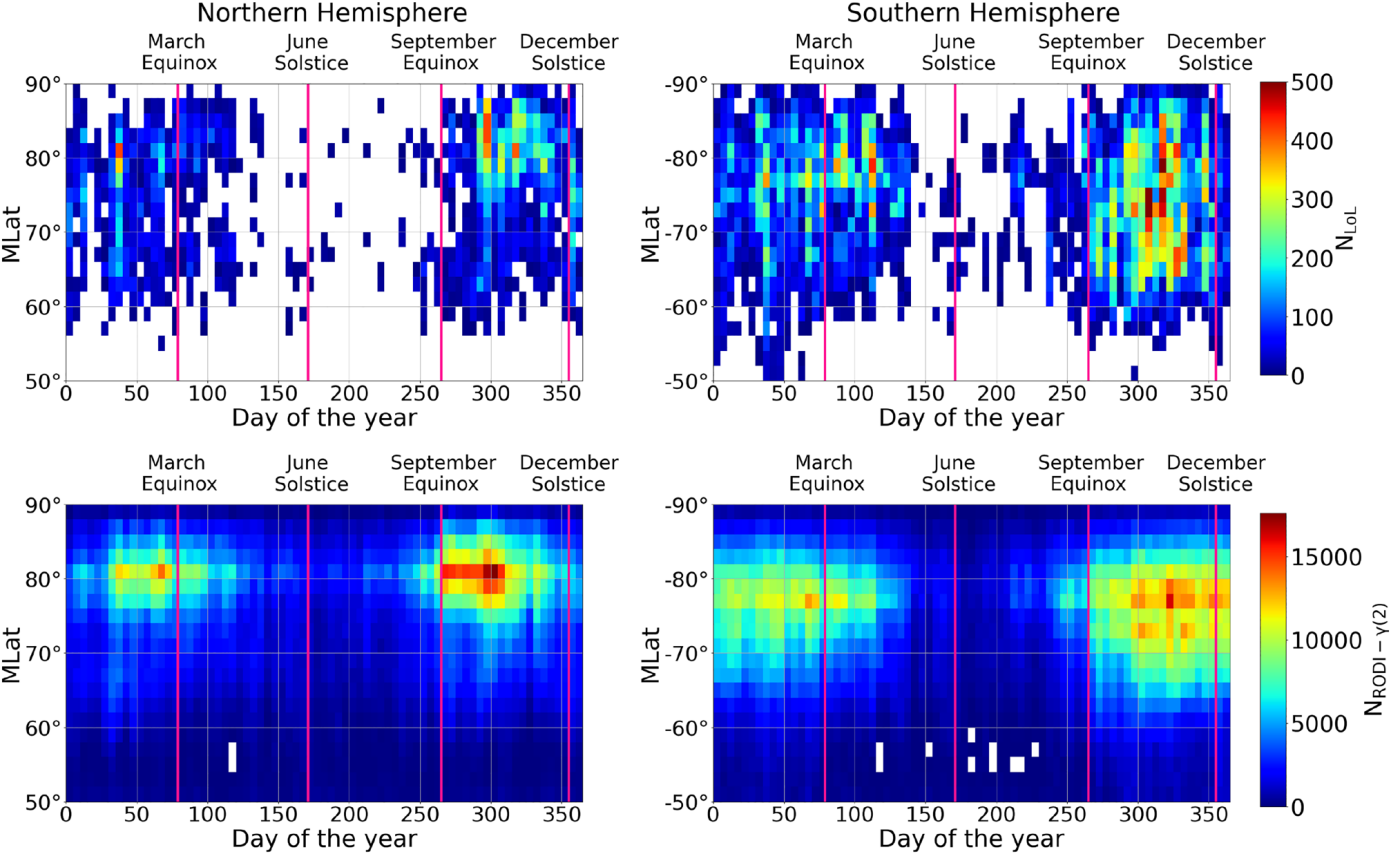
Distribution of GPS LoL events and $N_\mathrm{e}$ fluctuations with values of $\gamma (2)$ and RODI inside the ellipse of Fig. 2 as a function of MLat and day of the year for the Northern and Southern hemispheres. The distributions are obtained considering data recorded from July 15th, 2014 to December 31st, 2021.

In order to determine whether $N_\mathrm{e}$ fluctuations, which seem to be at the origin of GPS LoL events, could be utilized as a proxy for their occurrence, firstly we looked into their possible seasonal dependence. The purpose is to see if the two quantities exhibit the same annual trend. For this reason, Fig. 5 shows the distribution of both GPS LoL events and $N_\mathrm{e}$ fluctuations with values of $\gamma (2)$ and RODI inside the ellipse of Fig. 2 as a function of MLat and day of the year, in bins $2^{\circ }$-wide in MLat and 5-days wide. These trends were obtained by separating the two hemispheres and analyzing the data over the entire time period.

The GPS LoL events are limited to the months from September to May. Regardless of latitude, the months of June, July, and August have the lowest incidence of LoL events. $N_\mathrm{e}$ fluctuations with values of $\gamma (2)$ and RODI inside the ellipse of Fig. 2 show the same annual trend as the GPS LoL events. Indeed, the incidences are concentrated between September and May, while their number is incredibly low between June and August. Additionally, the regions of maximum occurrences appear wider and shifted at slightly lower latitudes in the Southern hemisphere than in the Northern one, most likely as a result of the different offset of the geographic and geomagnetic poles in the two hemispheres.

**Figure 6:**
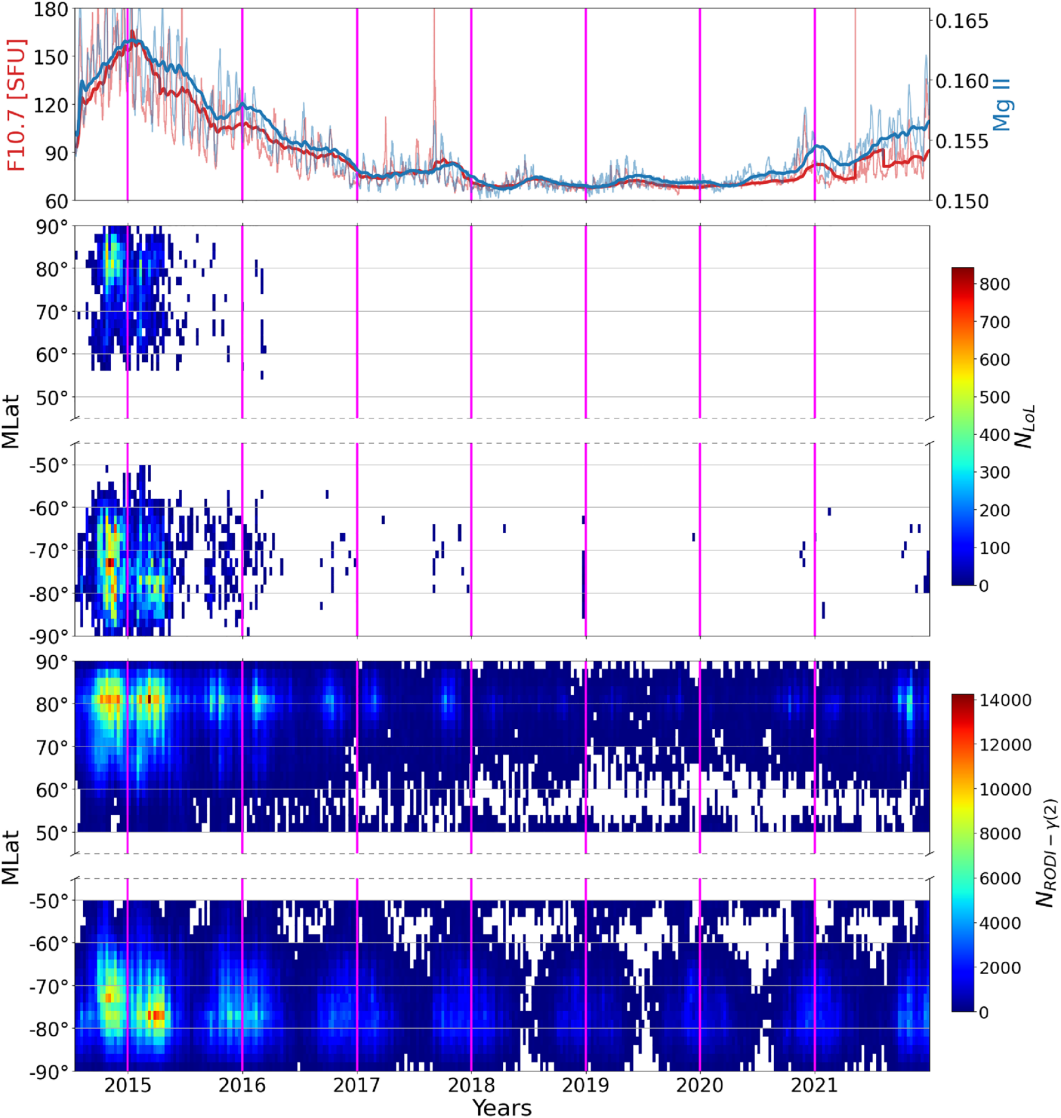
*First panel:* hourly values of the F10.7 solar index (thin red curve) and corresponding 81-day running mean (thick red curve), together with daily values of Mg II index (thin blue curve) and corresponding 81-days running mean (thick blue curve). *Second and Third panels:* distribution of GPS LoL events as a function of MLat and time, for the Northern and Southern hemisphere, respectively. *Fourth and Fifth panels:* distribution of $N_\mathrm{e}$ fluctuations with values of $\gamma (2)$ and RODI inside the ellipse of Fig. 2 as a function of MLat and time, for the Northern and Southern hemisphere, respectively. The time frame covered by all of the panels is from July 15th, 2014, to December 31st, 2021. The transition from one year to the next is represented by vertical magenta lines.

We also look at how LoL events and $N_\mathrm{e}$ fluctuations with values of $\gamma (2)$ and RODI inside the ellipse of Fig. 2 depend on solar activity. Figure 6 shows the evolution of the two distributions (second and third rows), as a function of MLat and day of the year, in bins $2^{\circ }$-wide in MLat and 9-days wide, over the solar cycle, as highlighted in the first row by two solar activity proxies: F10.7 (red curves) and Mg II core-to-wing ratio (blue curves). The first index refers to the radio emission at 10.7 cm wavelength, which originates from regions of intense magnetic field, characterized by structures like plages, chromospheric networks, and sunspots^36^. This index has been downloaded from the NASA OMNI Web Data Explorer website (https://omniweb.gsfc.nasa.gov/form/dx1.html). On the other side, Mg II index refers to the core-to-wing ratio of the Mg II line at 280 nm. The emission doublet in the line core originates in the Sun’s chromosphere, while the wings part originates in the photosphere, remaining much more steady over time. Thus, the ratio of line core intensity to wing intensity provides a robust estimate of solar variability, less susceptible to instrumental and degradation effects^37^. Moreover, this index has already shown its effectiveness when studying the ionosphere-thermosphere region response to solar activity^38–40^. Mg II index has been downloaded from UVSAT Bremen University dataset (https://www.iup.uni-bremen.de/UVSAT/Datasets/mgii, accessed on 29 November 2022). As seen in the second row, GPS LoL events are many during solar maximum years, namely in 2014 and 2015, and then they almost disappear in both hemispheres. Looking at the third row, one can see that the $N_\mathrm{e}$ fluctuations that meet the ellipse’s criterion tend to peak during periods of higher solar activity. In the following years of low solar activity, only a background remains, interspersed with evenly distributed minima corresponding to the period from June to August of each year, as shown in Fig. 5.

**Figure 7:**
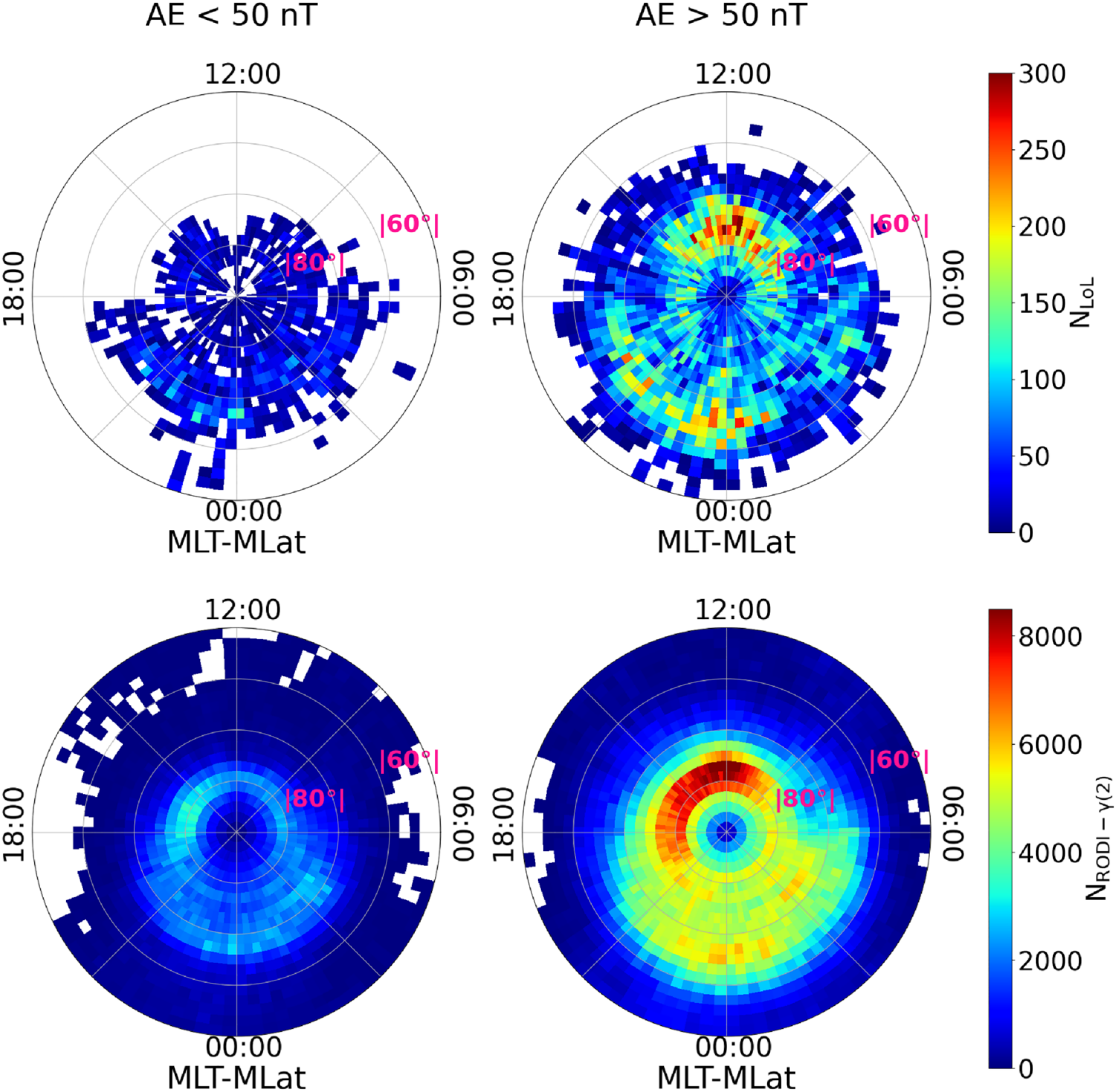
Comparison of the spatial distribution of GPS LoL events at mid and high latitudes ($|\textrm{MLat}|>50^{\circ }$) and the spatial distribution of $N_\mathrm{e}$ fluctuations with values for $\gamma (2)$ and RODI that are potentially capable of producing GPS LoL events during periods of quiet (left column) and disturbed (right column) geomagnetic activity conditions, respectively. In this case the datasets of both hemispheres have been joined.

Lastly we investigated the dependence of GPS LoL events and $N_\mathrm{e}$ fluctuations with values of $\gamma (2)$ and RODI inside the ellipse of Fig. 2 on geomagnetic activity, using the Auroral Electrojet (AE) index^41^. This index, which is available at 1 minute cadence, provides a global, quantitative measure of auroral zone magnetic activity, generated by the entire set of ionospheric currents flowing in the auroral zone. It was used to distinguish between two geomagnetic conditions in the dataset: quiet and disturbed. As a threshold between these two conditions, we used AE = 50 nT, which is the 25th percentile of the cumulative distribution during the chosen period. The results are displayed in Fig. 7. The quiet distributions are shown in the left column, while the disturbed distributions are shown in the right. In this case, the two hemispheres are not separated. This is because, as shown in Fig. 3, the Northern hemisphere presents fewer GPS LoL events, and separating them according to the aforementioned geomagnetic conditions makes it difficult to visualize the distribution, especially during quiet periods. As a result, we decide to join the two hemispheres in order to better visualize the global impact of geomagnetic activity. For both distributions the majority of events occur during disturbed geomagnetic conditions, namely $87\%$ for GPS LoL events and $77\%$ for the $N_\mathrm{e}$ fluctuations that meet the ellipse’s criterion. This confirms the significance of these events in the framework of space weather as solar activity appears to favor both LoL events as well as the formation of electron density irregularities that can trigger them.

## Discussion

Recently, research has been done on the distribution of Swarm LoL events^10,11^. The studies have revealed that these events are dependent on factors such as magnetic and geographic coordinates, season, and solar cycle. Additionally, it has been emphasized that the occurrence of LoL events is linked to high RODI values^11^. At the same time, it has been demonstrated that GPS LoL events are associated with a specific category of $N_\mathrm{e}$ fluctuations. These fluctuations are characterized by RODI values exceeding $10^4$ cm$^{-3}$ s$^{-1}$ and have second-order scaling exponents $\gamma (2)$ that suggest the presence of turbulent processes at their origin^7^. The aim of this study is to determine if the plasma conditions, that appear to be the cause of GPS LoL events, could also be used as a key indicator of their occurrence. For this reason we carefully investigated some of the characteristics and dependencies of LoL events, and then examined whether the $N_\mathrm{e}$ fluctuations characterized by the RODI and $\gamma (2)$ values typical of LoL events exhibited the same characteristics. Indeed, after determining the range of RODI and $\gamma (2)$ values that characterize $N_\mathrm{e}$ fluctuations during LoL events (see Fig. 2), we decided to investigate the relationship between the two data sets (LoL events and the corresponding class of fluctuations with specific RODI and $\gamma (2)$ values) as a function of magnetic latitude, magnetic local time, season, solar activity, and geomagnetic activity. The findings are very intriguing.

Firstly, in both hemispheres, the distributions of the two distinct data sets on the MLat-MLT plane agree well. Both LoL events and the corresponding class of fluctuations with specific RODI and $\gamma (2)$ values have a maximum occurrence at high latitude ($|\textrm{MLat}|> 60^{\circ }$), as shown in Fig. 3. LoL events, in particular, tend to occur preferentially in two regions: the magnetic cusp and the nightside auroral oval. A large number of $N_\mathrm{e}$ fluctuations with RODI and $\gamma (2)$ values typical of LoL events can also be found at the same locations. This result is visible in Fig. 4, where the two distributions are overlapped. What distinguishes the two distributions is not so much their location on the MLat-MLT plane, but rather the areas where their maximum values are observed (see Fig. 3). At the cusps, which are areas in the dayside auroral oval close to noon in the Northern and Southern high-latitude ionosphere, a maximum occurrence rate is clearly visible in both data sets. This area is within a few hours of MLT, and its location depends on how the interplanetary magnetic field is oriented. In our case, the cusp is nearly symmetrical with respect to noon and does not move before or after this, as it should when the interplanetary magnetic field’s orientation changes. Indeed, our distributions are derived by averaging about 7 years of data, without making any assumptions about the orientation of the interplanetary magnetic field, and thus have a climatological validity. The solar-wind condition changes have a direct impact on these regions, which are characterized by enhanced ion and electron precipitation. As confirmed by our findings, cusp processes can generate localized plasma density irregularities with a turbulent character, which can be also responsible for LoL occurrences. Another aspect to consider is that the number of events occurring in the Southern hemisphere is significantly greater than those occurring in the Northern hemisphere for both data sets. It was suggested that the larger number of ionospheric irregularities characterizing the Southern hemisphere could be related to the different offset of the geographic and geomagnetic poles in the two hemispheres, which would cause ionospheric convection in the polar cap of the Southern hemisphere to pull in more high-density plasma^42^. On the other hand, the difference between the two data sets is particularly noticeable on the night side, between $-80^{\circ }<$MLat$<-70^{\circ }$ in the Southern hemisphere, where $N_\mathrm{e}$ fluctuations with $\gamma (2)$ and RODI values that meet the ellipse criterion are maxima between 01:00 MLT and 05:00 MLT while LoL events tend to cluster between 18:00 MLT and 01:00 MLT. This suggests that while there are typical turbulent $N_\mathrm{e}$ fluctuations in these regions, LoL events tend to occur less frequently there, suggesting that the factors identified (RODI and $\gamma (2)$) are necessary but not sufficient. Moreover, an overall comparison of the distributions of the two data sets reveals that the number of LoL events is clearly lower than the number of $N_\mathrm{e}$ fluctuations presenting RODI and $\gamma (2)$ values compatible with the occurrence of LoL. The difference in the total number of events characterizing the two data sets is independent of the hemisphere under consideration. This implies that we may be able to track ionospheric conditions that favor GPS LoL events more effectively by introducing additional parameters that, in addition to those already identified, can better capture the $N_\mathrm{e}$ fluctuations at the base of LoL events. Anyhow, the good agreement between the two distributions is encouraging and demonstrates the importance of this class of RODI-$\gamma (2)$ values in assessing the occurrence of GPS LoL events.

Very interesting is the result obtained analysing the annual occurrence rate of the two data sets. The results reveal that there is no true seasonal dependence, but rather a lower occurrence rate in the months surrounding the June solstice in both hemispheres, regardless of the corresponding local season. At very high latitude ($|\textrm{MLat}|> 75^{\circ }$), it seems to be consistent with the annual occurrence rate of the polar cap patches, which are plasma density irregularities with densities that are at least twice as high as the surrounding plasma^43^. Indeed, it has been observed that polar cap patches are more in the Northern hemisphere in winter than in summer, while in the Southern hemisphere Noja et al. (2013)^44^, using the upward-looking GPS TEC data from CHAMP, identified more patches in the summer Southern hemisphere. It should be noted that Chartier et al. (2018)^45^ found a different result using electron density data collected by the Swarm satellite constellation, concluding that polar cap patches are more prevalent during local winter in both hemispheres, whereas the same investigation using upward-looking TEC revealed a completely different result: polar cap patches appear to occur more frequently in both hemispheres in December. The method of identifying the polar cap patches, which is based on in situ density measurement or integrated TEC, may be the cause of the discrepancy. Our findings suggest that $N_\mathrm{e}$ fluctuations that meet the ellipse criterion may be associated with polar cap patches at very high latitudes, which may also be partially responsible for LoL events. The impact of solar extreme ultraviolet (EUV) radiation, which ionizes the E region during the summer and may short-circuit the F-region irregularities, can explain this cyclical trend in the Northern hemisphere. Because this argument is no longer valid in the Southern Hemisphere, alternative processes should be investigated to explain the apparent reduction of irregularities between June and August. It is possible that it is related to the ionospheric annual anomaly, which is the fact that the global maximum of electron density appears higher in December than in June.

The two distributions exhibit very similar behavior even when studying their dependence on solar activity, peaking during the same year of increased solar activity. Nevertheless, while GPS LoL events almost disappear during solar minimum, a background exists for $N_\mathrm{e}$ fluctuations with values of $\gamma (2)$ and RODI that meet the ellipse criterion. Anyway, this reduction in the occurrences of GPS LoL events and $N_\mathrm{e}$ fluctuations with RODI and $\gamma (2)$ inside the ellipse with the lowering of solar activity, follows the previously observed behavior of ionospheric irregularities, regardless of latitude^46,47^. During periods of increased solar activity, irregularities in the ionospheric F-region occur against a background of increased ionization density^5^. This is also evident in the work of Jin and coauthors^42^, which shows the annual trend of three parameters related to the presence of ionospheric irregularities from 2014 to 2018, namely the electron density gradient $\nabla N_e$, the RODI, and the ROTI, which represents the standard deviation of the TEC time derivative. The values of these irregularity parameters decrease significantly at all locations (cusp, polar cap, and nightside auroral oval) as the solar cycle declines, which corresponds to an electron density background decrease.

Finally, the dependence on geomagnetic activity level showed that disturbed periods favor both $N_\mathrm{e}$ fluctuations that satisfy the ellipse’s criterion and GPS LoL events. During geomagnetic disturbed periods, which were chosen based on auroral electrojet index values $\textrm{AE} > 50$ nT, the area covered by the two data sets tends to be larger than that covered during quiet periods, reaching lower magnetic latitudes. The number of events increases dramatically in correspondence with the cusp and on the night side. Particularly on the night side, the region affected by both LoL events and plasma fluctuations with values of $\gamma (2)$ and RODI satisfying the ellipse criterion reaches $\sim |60^{\circ }|$ of magnetic latitude between 21:00 and 03:00 MLT. With regard to this region, the Main Ionospheric Trough (MIT) might play a role in the development of ionospheric irregularities and GPS LoL events. In fact, this structure lies typically between the footprints of the plasmapause/plasmaspheric boundary layer^48,49^ and the equatorward boundary of the auroral oval. It can have a significant impact on the propagation of radio signals and, consequently, on the GPS signal because of the resulting large density gradient characterizing the area. It has been showed^50^ that the trough depth exhibits a clear increasing trend with increasing geomagnetic activity, as tracked by the Kp index value, similarly to what we found for GPS LoL events and for $N_\mathrm{e}$ fluctuations that satisfy the ellipse’s criterion. Anyway, this link should be further examined because the other MIT climatological dependencies reported by Aa et al. (2020)^50^ agree only partially with the ones of our distributions. However, the comparison is not straightforward because their analysis is done considering only quiet geomagnetic conditions when examining the dependencies of the MIT on magnetic coordinates, local season and solar activity. Anyhow, the number of LoL events and the number of $N_\mathrm{e}$ fluctuations with a turbulent character are high during disturbed geomagnetic period, suggesting that they are relevant in the space weather framework. This is also supported by the already mentioned dependence on solar activity. In fact, the ionospheric activity in polar, sub-auroral, and mid-latitude regions is primarily associated with geomagnetic storms and substorms, coronal mass ejections, and coronal holes^51^ and the GPS LoL events, as well as the turbulent irregularities associated with them, seem to be no exception.

In conclusion, our research highlights that the $N_\mathrm{e}$ fluctuations (Fig. 2), which define a specific family of values in the RODI-$\gamma (2)$ space, have spatio-temporal dependencies that are very similar to those of GPS LoL events. As a result, identifying this specific class of turbulent ionospheric fluctuations might play a key role in the future development of GPS LoL hazard maps for the mid- and high-latitude region, and thus contribute significantly to the contest of space weather effect mitigation. However, since the number of LoL events actually recorded is clearly lower than the number of events in which conditions favorable to their occurrence develop, further research is necessary to make an even better selection of fluctuations with properties compatible with those of the occurrence of LoL events.

## Methods

One of the most notable characteristics of turbulent signals is the scale-invariant nature of their fluctuations, which denotes the lack of a distinctive scale for the emergent fluctuation structures. An easy technique to determine whether scaling features appear in a time series is to evaluate the so-called generalized $q^{\textrm{th}}$-order structure function $S_q$^52^, which is defined as:

1 $$\begin{aligned} S_q(t) = \langle \mid f(t+\delta t)-f(t)\mid ^q\rangle , \end{aligned}$$

where *f*(*t*) is the time-series, $\delta t$ is the temporal increment, and $\langle ...\rangle$ stands for a statistical mean. The $q^{\textrm{th}}$-order structure function is predicted to scale as follows for scale-invariant signals:

2 $$\begin{aligned} S_q(t) =\delta t ^{\gamma (q)}, \end{aligned}$$

where $\gamma (q)$ is the $q^{\textrm{th}}$-order scaling exponent. The spectral characteristics of the fluctuations are revealed by the second-order scaling exponent, $\gamma (2)$, as stated by the Wiener-Khinchin theorem^53^. In fact, the relationship $\beta = \gamma (2) +1$ exists between the scaling exponent of the second-order structure function and the exponent of the power spectral density, $\beta$. Therefore, the knowledge of the local scaling properties can be used to indirectly infer the spectral features of the analyzed time-series. These, in turn, allow differentiating the various types of instabilities or turbulent processes that cause the observed fluctuations. We used the detrended structure function analysis (DSFA) method^54^ to evaluate the second-order scaling exponent. In the past, it has been successfully used to look into the local scaling characteristics of the electron density^13^ and magnetic field fluctuations^54–56^, derived using equipments aboard the Swarm constellation. The core of the DSFA method is the application of structure function analysis to locally detrended time series, in order to evaluate local scaling exponents within moving windows. In this case, we took into account a window of 301 points and evaluated the structure function of $N_\mathrm{e}$ for each window. In this manner, the second-order scaling exponent values, which are scaling characteristics of the local $N_\mathrm{e}$ fluctuations, were determined. The choice of a window of 301 points corresponds to analyze the features of $N_\mathrm{e}$ fluctuations over a spatial range of about 2400 km, due to the Swarm constellation’s orbital speed of about 8 km/s. Additionally, since the satellite passes through regions where various physical processes occur, this window size appeared to be a good compromise for avoiding their mixing. We analyzed time increments, $\delta t$, between 1 and 40 seconds inside each window, and we linked the estimated second-order scaling exponents to the satellite’s position, that corresponds to the window’s center. This means that we analyzed spatial increments from 8 km to 320 km, under the hypothesis that the observed structure’s transit time is faster than its evolution^57^. Since several processes and phenomena that are typical of the ionosphere at high and low latitudes occur in the meso-scale domain (i.e., between tens and hundreds of km), the analyzed spatial scales can be particularly interesting to study the characteristics of $N_\mathrm{e}$ fluctuations occurring there. In fact, at these scales, it is possible to see the effects of some processes and/or phenomena including polar cap patches, blobs, auroral arcs, field-aligned currents, and plasma bubbles. Although the dataset used has a limited temporal resolution, it is possible that the properties of electron density fluctuations observed at the mesoscale level may also be present at smaller scales. The turbulence mechanism that drives these fluctuations should extend to smaller scales within the magnetohydrodynamic domain, as demonstrated by previous studies^3,15,58^. Therefore, it is important to consider the possibility that smaller scales may also exhibit similar properties, despite the current limitations in analyzing them.

## Data Availability

Swarm data can be accessed online (https://swarm-diss.eo.esa.int/), as well as OMNI data (https://cdaweb.gsfc.nasa.gov/index.html) and Mg II index data (https://www.iup.uni-bremen.de/UVSAT/Datasets/mgii).

## References

[1] Moldwin M (2008). An Introduction to Space Weather.

[2] Kintner PM, Kil H, Beach TL, de Paula ER (2001). Fading timescales associated with gps signals and potential consequences. Radio Sci..

[3] Basu S (1988). Simultaneous density and electric field fluctuation spectra associated with velocity shears in the auroral oval. J. Geophys. Res..

[4] Jin Y, Moen JI, Miloch WJ (2014). Gps scintillation effects associated with polar cap patches and substorm auroral activity: Direct comparison. J. Space Weather Space Clim..

[5] Basu S, Groves K, Basu S, Sultan P (2002). Specification and forecasting of scintillations in communication/navigation links: Current status and future plans. J. Atmos. Solar Terr. Phys..

[6] Jin Y (2020). Ionospheric plasma irregularities based on in situ measurements from the swarm satellites. J. Geophys. Res. Space Physics.

[7] De Michelis P (2022). Ionospheric turbulence: A challenge for GPS loss of lock understanding. Space Weather.

[8] Buchert S (2015). Swarm observations of equatorial electron densities and topside gps track losses. Geophys. Res. Lett..

[9] Xiong C, Stolle C, Lühr H (2016). The Swarm satellite loss of GPS signal and its relation to ionospheric plasma irregularities. Space Weather.

[10] Xiong C, Stolle C, Park J (2018). Climatology of GPS signal loss observed by Swarm satellites. Ann. Geophys..

[11] Pezzopane M (2021). Occurrence of GPS loss of lock based on a swarm half-solar cycle dataset and its relation to the background ionosphere. Remote Sens..

[12] Earle G, Kelley M, Ganguli G (1989). Large velocity shears and associated electrostatic waves and turbulence in the auroral f region. J. Geophys. Res. Space Phys..

[13] Giannattasio, F. *et al.* Characterising the electron density fluctuations in the high-latitude ionosphere at swarm altitude in response to the geomagnetic activity. *Ann. Geophys.* **62**. 10.4401/ag-7790 (2019).

[14] Cerisier J, Berthelier J, Beghin C (1985). Unstable density gradients in the high-latitude ionosphere. Radio Sci..

[15] Mounir H, Berthelier A, Cerisier JC, Lagoutte D, Beghin C (1991). The small-scale turbulent structure of the high latitude ionosphere - Arcad-Aureol-3 observations. Ann. Geophys..

[16] Basu S (1990). Plasma structuring by the gradient drift instability at high latitudes and comparison with velocity shear driven processes. J. Geophys. Res. Space Phys..

[17] Carlson, H. C., Pedersen, T., Basu, S., Keskinen, M. & Moen, J. Case for a new process, not mechanism, for cusp irregularity production. *J. Geophys. Res. Space Phys.* **112** (2007).

[18] Tsunoda RT, Haggstrom I, Pellinen-Wannberg A, Steen A, Wannberg G (1985). Direct evidence of plasma density structuring in the auroral F region ionosphere. Radio Sci..

[19] Kolmogorov AN (1941). The local structure of turbulence in incompressible viscous fluid for very large reynolds numbers. Cr Acad. Sci. URSS.

[20] Dyson P, McClure J, Hanson W (1974). In situ measurements of the spectral characteristics of f region ionospheric irregularities. J. Geophys. Res..

[21] Tsunoda RT (1988). High-latitude F-region irregularities: A review and synthesis. Rev. Geophys..

[22] Kivanc O, Heelis RA (1998). Spatial distribution of ionospheric plasma and field structures in the high-latitude f region. J. Geophys. Res. Space Phys..

[23] Spicher A, Miloch WJ, Moen JI (2014). Direct evidence of double-slope power spectra in the high-latitude ionospheric plasma. Geophys. Res. Lett..

[24] Di Mare, F., Spicher, A., Clausen, L. B. N., Miloch, W. J. & Moen, J. I. Turbulence and intermittency in the winter cusp ionosphere studied with the ICI sounding rockets. *J. Geophys. Res. (Space Phys.)* **126**, e29150. 10.1029/2021JA029150 (2021).

[25] De Michelis P (2021). Looking for a proxy of the ionospheric turbulence with Swarm data. Sci. Rep..

[26] De Michelis P (2021). Ionospheric turbulence and the equatorial plasma density irregularities: Scaling features and RODI. Remote Sens..

[27] Knudsen DJ (2017). Thermal ion imagers and Langmuir probes in the Swarm electric field instruments. J. Geophys. Res. (Space Phys.).

[28] van den IJssel, J., Encarnação, J., Doornbos, E. & Visser, P. Precise science orbits for the Swarm satellite constellation. *Adv. Space Res.* **56**, 1042–1055. 10.1016/j.asr.2015.06.002 (2015).

[29] Friis-Christensen E, Lühr H, Knudsen D, Haagmans R (2008). Swarm an earth observation mission investigating. Geospace Adv. Space Res..

[30] Pignalberi A (2021). TITIPy: A Python tool for the calculation and mapping of topside ionosphere turbulence indices. Comput. Geosci..

[31] Swarm L2 TEC Product Description (2017). https://earth.esa.int/eogateway/documents/20142/37627/swarm-level-2-tec-product-description.pdf/8fe7fa04-6b4f-86a7-5e4c-99bb280ccc7e.

[32] Richmond AD (1995). Ionospheric electrodynamics using magnetic apex coordinates. J. Geomagn. Geoelectr..

[33] Emmert, J. T., Richmond, A. D. & Drob, D. P. A computationally compact representation of Magnetic-Apex and Quasi-Dipole coordinates with smooth base vectors. *J. Geophys. Res.* **115**. 10.1029/2010JA015326 (2010).

[34] Laundal KM, Richmond AD (2017). Magnetic coordinate systems. Space Sci. Rev..

[35] De Michelis, P. *et al.* On the 2015 St. Patrick’s Storm Turbulent State of the Ionosphere: Hints From the Swarm Mission. *J. Geophys. Res. (Space Phys.)* **125**, e27934. 10.1029/2020JA027934 (2020).

[36] Foukal P (1998). Extension of the f10. 7 index to 1905 using mt. wilson ca k spectroheliograms. Geophys. Res. Lett..

[37] Peeters, P. *et al.* Mg ii core-to-wing solar index from high resolution gome data. In *ESA-SP 414: Proceedings of the 3rd ERS Symposium ’Space at the Service of our Environment’*, vol. 414, 719–722 (ESA, 1997).

[38] Perna L, Pezzopane M (2016). fof2 vs solar indices for the rome station: Looking for the best general relation which is able to describe the anomalous minimum between cycles 23 and 24. J. Atmos. Solar Terr. Phys..

[39] Vaishnav R, Jacobi C, Berdermann J (2019). Long-term trends in the ionospheric response to solar extreme-ultraviolet variations. Ann. Geophys..

[40] Bigazzi A, Cauli C, Berrilli F (2020). Lower-thermosphere response to solar activity: an empirical-mode-decomposition analysis of GOCE 2009–2012 data. Ann. Geophys..

[41] Davis TN, Sugiura M (1966). Auroral electrojet activity index ae and its universal time variations. J. Geophys. Res..

[42] Jin Y (2019). Ionospheric plasma irregularities characterized by the swarm satellites: Statistics at high latitudes. J. Geophys. Res. Space Physics.

[43] Crowley G (1996). Critical review of ionospheric patches and blobs. Rev. Radio Sci..

[44] Noja M, Stolle C, Park J, Lühr H (2013). Long-term analysis of ionospheric polar patches based on CHAMP TEC data. Radio Sci..

[45] Chartier AT, Mitchell CN, Miller ES (2018). Annual occurrence rates of ionospheric polar cap patches observed using swarm. J. Geophys. Res. Space Phys..

[46] Aarons J, Mullen J, Whitney H, Johnson A, Weber E (1981). Uhf scintillation activity over polar latitudes. Geophys. Res. Lett..

[47] Rodger AS, Graham AC (1996). Diurnal and seasonal occurrence of polar patches. Ann. Geophys..

[48] Carpenter D, Lemaire J (2004). The Plasmasphere Boundary Layer. Ann. Geophys..

[49] Pierrard, V. & Voiculescu, M. The 3d model of the plasmasphere coupled to the ionosphere. *Geophys. Res. Lett.* **38** (2011).

[50] Aa E, Zou S, Erickson PJ, Zhang S-R, Liu S (2020). Statistical analysis of the main ionospheric trough using swarm in situ measurements. J. Geophys. Res. Space Phys..

[51] Cherniak I, Zakharenkova I, Krankowski A (2014). Approaches for modeling ionosphere irregularities based on the tec rate index. Earth Planets Space.

[52] Frisch, U. *Turbulence: The Legacy of A. N. Kolmogorov* (Cambridge University Press, 1995).

[53] Wiener, N. (ed.) *Time Series* (M.I.T. Press, 1964).

[54] De Michelis P, Consolini G, Tozzi R (2015). Magnetic field fluctuation features at Swarm’s altitude: A fractal approach. Geophys. Res. Lett..

[55] De Michelis, P., Consolini, G., Tozzi, R. & Marcucci, M. F. Observations of high-latitude geomagnetic field fluctuations during St. Patrick’s Day storm: Swarm and SuperDARN measurements. *Earth Planets Space* **68**, 105. 10.1186/s40623-016-0476-3 (2016).

[56] De Michelis P, Consolini G, Tozzi R, Marcucci MF (2017). Scaling Features of High-Latitude Geomagnetic Field Fluctuations at Swarm Altitude: Impact of IMF Orientation. J. Geophys. Res. (Space Phys.).

[57] Taylor, G. I. The spectrum of turbulence. *Proc. R. Soc. Lond. Ser. A Math. Phys. Sci.* **164**, 476–490. 10.1098/rspa.1938.0032 (1938). https://royalsocietypublishing.org/doi/pdf/10.1098/rspa.1938.0032.

[58] Spicher A, Miloch W, Clausen L, Moen J (2015). Plasma turbulence and coherent structures in the polar cap observed by the ICI-2 sounding rocket. J. Geophys. Res. Space Phys..

